# How Do Oriental Reed Warblers Recognize Cuckoo Eggs?

**DOI:** 10.1002/ece3.72664

**Published:** 2025-12-14

**Authors:** Hanlin Yan, Longwu Wang, Wei Liang

**Affiliations:** ^1^ School of Biological Science and Technology Liupanshui Normal University Liupanshui China; ^2^ Ministry of Education Key Laboratory for Ecology of Tropical Islands, Key Laboratory of Tropical Animal and Plant Ecology of Hainan Province, College of Life Sciences Hainan Normal University Haikou China

**Keywords:** *Acrocephalus orientalis*, cognitive mechanism, *Cuculus canorus*, egg recognition, host

## Abstract

Oriental reed warblers ( *Acrocephalus orientalis* ) are favorite hosts of common cuckoos ( *Cuculus canorus* ). However, the cognitive basis and underlying mechanisms of the host egg discrimination behavior remain not well understood. In this study, we conducted egg recognition experiments using three egg mimicry levels (non, poorly, and highly mimetic eggs) to observe Oriental reed warbler behavior (rejection or acceptance) at three breeding stages (pre‐egg‐laying, single host egg, and multiple host eggs). Results showed that oriental reed warblers accepted all highly‐mimetic (conspecific) eggs across all stages, while rejecting most non‐ and poorly mimetic eggs. Rejection rates for nonmimetic eggs were 100% in both pre‐egg‐laying and single‐egg stages, and 86.7% in the multiple‐egg stage; for poorly mimetic eggs, rejection rates were 83.3%, 70%, and 66.7%, respectively. The ability to reject foreign eggs even at the pre‐egg‐laying stage suggests that oriental reed warblers may use a memory‐based template for egg recognition, relying on innate or experientially acquired cues. However, the consistent acceptance of conspecific eggs also aligns with uncertainty‐based acceptance strategies. These findings indicate that oriental reed warblers employ a complex and potentially multi‐mechanistic cognitive system for egg recognition.

## Graphical abstract

The cognitive basis and underlying mechanisms of the host egg rejection behavior remain not well understood. Egg recognition experiments using three egg mimicry levels (non, poorly, and highly mimetic eggs) to observe Oriental reed warbler ( *Acrocephalus orientalis* ) behavior at three breeding stages (pre‐egg‐laying, single host egg, and multiple host eggs). Recognition and rejection of most foreign eggs, but none of the conspecific eggs, at the pre‐egg‐laying stage without any comparison indicates true egg recognition in response to parasitism.

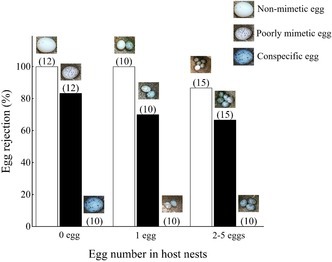

## 1 Introduction

Avian brood parasitism is a specialized reproductive behavior within avian life history strategies; it refers to the practice where parasitic birds lay their eggs in the nests of host birds during the breeding season, compelling the hosts to incubate and raise the parasitic offspring (Payne 1977; Davies 2000). This allows the parasites to reduce the reproductive costs associated with raising their own young (Payne 1977; Davies 2000; Soler 2014). Egg recognition is the behavior by which hosts identify their own versus foreign eggs in their nests (Rothstein 1990). Recognition and rejecting foreign eggs in their nests by bird hosts is a specific antiparasitic adaptation and behavioral response to parasitism (Davies 2000; Langmore et al. 2005). Egg rejection behaviors by hosts promote the evolution of egg mimicry in parasites (Brooke and Davies 1988). Through coevolution, the hosts gradually improve their egg recognition abilities, and the parasites improve their egg mimicry abilities (Soler 2014; Xu et al. 2023). For example, after studying the spectral and pattern polymorphism of eggs laid by the parasitic cuckoo‐finch ( *Anomalospiza imberbis* ) and its hosts in Africa, Spottiswoode and Stevens (2010) used avian visual modeling to show that rejection behavior is predicted by disparities in multiple egg traits, including color, pattern dispersion, marking size, and the dominance of a single marking size. These cues correspond to the traits that differ most between host and parasitic eggs, illustrating how hosts use reliable visual signals to detect parasitism and thereby drive the evolution of egg mimicry in parasites.

The hosts' process of parasitic egg rejection generally includes at least three stages. The first is recognizing a foreign egg, the second is deciding whether to reject the egg, and the third stage is actually rejecting and disposing of the egg (Moskát and Hauber 2007; Soler et al. 2012, 2017). However, the form and extent of egg rejection behaviors vary among host species, and even among populations of the same host species (Hauber and Sherman 2001; Moskát and Honza 2002; Yang et al. 2015). Each host exhibits varying responses to different types of egg mimicry depending on the conditions (Avilés et al. 2005; Holen and Johnstone 2006; Servedio and Hauber 2006). This suggests that egg rejection behaviors exhibit both consistency and flexibility and that genetic and learning mechanisms may be involved (Lotem et al. 1995; Hauber and Sherman 2001; Martín‐Gálvez et al. 2006; Moskát et al. 2010; Wang et al. 2015). To date, four possible mechanisms have been proposed to explain egg recognition behaviors to counter brood parasitism: (i) Direct comparison: hosts compare eggs within the same clutch and reject those that differ from the majority (Rothstein 1975; Moskát et al. 2010). This mechanism requires the presence of multiple eggs for effective discrimination. (ii) Memory‐based template: hosts rely on an innate or experientially acquired mental representation of their own egg phenotype to identify foreign eggs, even in the absence of their own eggs (Lotem et al. 1995; Hauber and Sherman 2001). For example, great reed warblers ( *Acrocephalus arundinaceus* ) can reject nonmimetic eggs during the pre‐laying stage, suggesting the use of a long‐term memory template (Moskát and Hauber 2007). This mechanism is particularly relevant when hosts encounter parasitic eggs before laying their own. (iii) Onset of laying: hosts reject any egg present in the nest before they begin laying their own clutch, regardless of its appearance (Davies 2000). This is a conservative strategy that minimizes the risk of parasitism but may lead to unnecessary rejection of conspecific eggs if applied after laying begins. (iv) Phenotype distribution: hosts reject eggs that fall outside the acceptable phenotypic range of their own egg traits, based on a learned or innate distribution of egg characteristics (Servedio and Lande 2003; Stokke et al. 2004). This mechanism allows for flexibility in acceptance thresholds depending on environmental or experiential factors.

However, recent syntheses have grouped these various mechanisms into two primary cognitive frameworks: template‐based recognition and discordancy‐based recognition (Manna et al. 2018; Yang et al. 2014). Template‐based recognition involves hosts comparing eggs to an internal representation (innate or learned) of their own eggs, enabling rejection even in the absence of their own eggs (Lotem et al.1995; Hauber and Sherman 2001). In contrast, discordancy‐based recognition involves rejecting the egg that differs most from the majority in the clutch, without requiring a memorized template (Rothstein 1975; Servedio and Hauber 2006; Moskát et al. 2010). These mechanisms are not mutually exclusive and can operate in tandem, with contextual factors such as breeding stage or parasitism frequency modulating their expression (Moskát et al. 2010; Yang et al. 2014). In this study, we focus on these two principal mechanisms to investigate the egg recognition behavior of the oriental reed warbler, a frequent host of the common cuckoo, across different breeding stages.

This study aimed to examine the cognitive mechanisms underlying the behaviors of the oriental reed warbler ( *Acrocephalus orientalis* , ORW) for recognizing parasitic eggs. ORWs are common hosts of the common cuckoo ( *Cuculus canorus* , CC), an avian obligate brood parasite, with parasitism rates reaching 34.3%–65.5% in northern China (Li et al. 2015; Wang et al. 2020). ORWs are generally considered rejectors of nonmimetic and poorly mimetic eggs, but evidence suggests that many host species may accept highly mimetic eggs or under conditions of perceptual uncertainty (Yang et al. 2016; Wang et al. 2015). Cuckoo eggs parasitizing ORWs exhibit moderate visual mimicry, though they often differ in spotting pattern and color intensity (Li et al. 2015). Previous studies have shown that ORWs employ multiple defensive strategies, including egg rejection, nest defense, and nest sanitation (Li et al. 2015; Yang et al. 2016), making them an ideal model for studying cognitive mechanisms of egg discrimination in response to brood parasitism (Ma and Liang 2021; Yang et al. 2015).

We conducted egg recognition experiments using three egg mimicry levels (non, poorly, and highly mimetic eggs) to observe ORW behavior at three breeding stages (pre‐egg‐laying, single host egg, and multiple host eggs). The “pre‐egg‐laying” stage can be used to test the “memory‐based template hypothesis”, the “single‐egg stage” can assess whether hosts rely on their own first egg as a reference, while the “multiple‐egg stage” is suitable for testing the “direct comparison” mechanism, where hosts identify foreign eggs by comparing the majority phenotype within the clutch (Moskát and Hauber 2007; Wang et al. 2015). By introducing experimental eggs with varying degrees of mimicry during these three phases, we can distinguish whether hosts rely on intrinsic templates, laying‐time signal cues, or decisions based on egg cluster phenotype frequencies. Among these, the memory‐based template hypothesis is especially relevant for hosts that encounter parasitic eggs prior to laying, as it allows for discrimination without concurrent reference eggs (Wang et al. 2015). In particular, the ORW often experiences parasitism before laying its own clutch (Li et al. 2015), making a memory‐based mechanism a likely candidate for egg discrimination. We hypothesized that if ORWs use memory‐based template for egg recognition, they would reject most non‐ and poorly mimetic foreign parasitic eggs but accept highly‐mimetic (conspecific) eggs during all three stages. However, if ORWs use the onset of laying for egg recognition, they would reject all eggs in the pre‐egg‐laying (Davies 2000), regardless of egg mimicry. If ORWs rely on a direct comparison mechanism, we predicted that rejection of foreign eggs would be most effective during the multiple‐egg stage, when hosts can compare eggs within the clutch and identify the one that differs from the majority (Rothstein 1975; Moskát et al. 2010). Under this mechanism, rejection rates for non‐ and poorly mimetic eggs should be highest in the multiple‐egg stage compared to earlier stages. Alternatively, if ORWs use a phenotype distribution mechanism, they would reject eggs falling outside the acceptable range of conspecific egg variation (Servedio and Lande 2003; Stokke et al. 2004).

## 2 Materials and Methods

The Sifangtuozi Farm (46°00′–46°22′ N, 123°46′–123°57′ E) is located in the Zhenlai County section of the Nenjiang River Basin. The area is under the jurisdiction of Jilin Province in Northeast China and has abundant water resources. Vast patches of reeds and bulrushes grow along the ridges, aqueducts, creeks, ponds, and other areas around the farmlands. During the annual breeding season (May–August), numerous ORWs migrate to the reed marshes in this area to breed (Trnka et al. 2023; Yan and Liang 2025). ORWs are the main hosts of CCs in Asia. The CC parasitism rate in northern China is as high as 34.3%–65.5% (Li et al. 2015; Wang et al. 2020, 2021, 2022; Yang et al. 2016, 2017).

We searched for new nests in the reed swamps at least twice a week from May to August 2022. Upon identification, the status of the nests was checked every morning. The ORWs of this population typically lay eggs in the early mornings, whereas CCs can lay eggs at any time of day.

**FIGURE 1:**
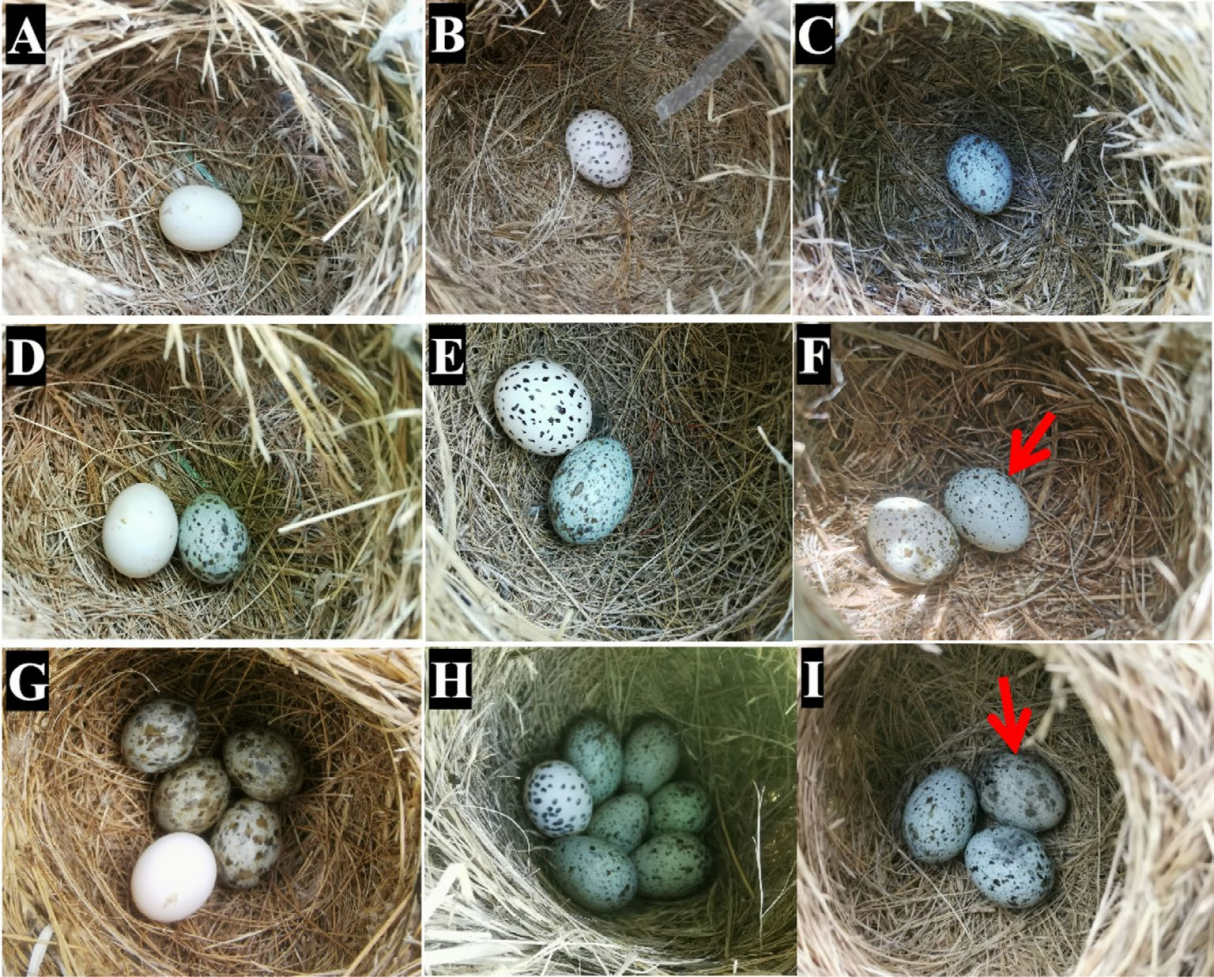
Experiments of egg recognition mechanisms (A–C) refer to nonmimetic egg, poorly mimetic eggs, and highly‐mimetic (conspecific) eggs in the pre‐egg‐laying stage; (D–F) refer to non‐, poorly and highly mimetic (shown by the red arrow) eggs in the one‐host egg stage; (G–I) refer to non, poorly and highly mimetic (shown by the red arrow) eggs in the multi‐host egg stage.

With reference to the methods of Moskát and Hauber (2007) and Wang et al. (2015), we used the eggs of the budgerigar ( *Melopsittacus undulatus* ) as the foreign eggs and ORW eggs from the same breeding population as the highly mimetic (conspecific) eggs. The experimental groups were established by removing one of the host eggs from their nest and replacing it with the following: (i) budgerigar egg (nonmimetic egg group; *n* = 37 nests, Figure 1A,D,G); (ii) budgerigar egg with black spots painted on the surface (poorly mimetic egg group; *n* = 37 nests, Figure 1B,E,H); and (iii) highly mimetic (conspecific egg group; *n* = 30 nests, Figure 1C,F,I).

Next, the three experimental groups were further subdivided into the following stages: (i) pre‐egg‐laying: *n* = 12 for nonmimetic egg and poorly mimetic egg groups, and *n* = 10 for highly‐mimetic group; (ii) single host egg: *n* = 10 for all groups; and (iii) multiple host eggs: *n* = 15 for nonmimetic egg and poorly mimetic egg groups, and *n* = 10 for highly‐mimetic group. For the pre‐egg‐laying stage (Figure 1A–C), an experimental egg was placed into each nest after nest building was completed but before egg‐laying. For the single host egg stage (Figure 1D–F), an experimental egg was introduced to replace the only host egg in the nest 1 day after the host had laid the egg. For the multiple host eggs stage (Figure 1G–I), an experimental egg was introduced to replace one of the host eggs after the host had laid 2–6 eggs (the maximum clutch size for ORWs is six eggs, see Figure 1H). To reduce human interference, we consistently limited our operation time to the morning after the host had laid the egg; that is, we tried our utmost to replace the host egg as soon as possible.

All experimental parasitism was conducted during the host's laying period, including the “multiple host eggs” stage. This ensures that behavioral responses are not confounded by reduced antiparasitic vigilance during incubation, as hosts are known to be less responsive to parasitic eggs later in the breeding cycle (Moskát, Hauber, et al. 2014; Abolins‐Abols and Hauber 2020).

We monitored the nests under the parasitism experiments daily for six consecutive days to confirm the hosts' behavioral responses. The status was classified as rejection if the experimental eggs were ejected or buried or the nests were abandoned (due to small sample sizes, we could not parse out nest abandonment and egg ejection/burial into their own classifications); the status was classified as acceptance if the experimental eggs remained intact in the host nest after six days or were hatched (Moksnes et al. 1991; Moskát and Hauber 2007; Wang et al. 2015).

### 2.1 Statistical Analyzes

**FIGURE 2:**
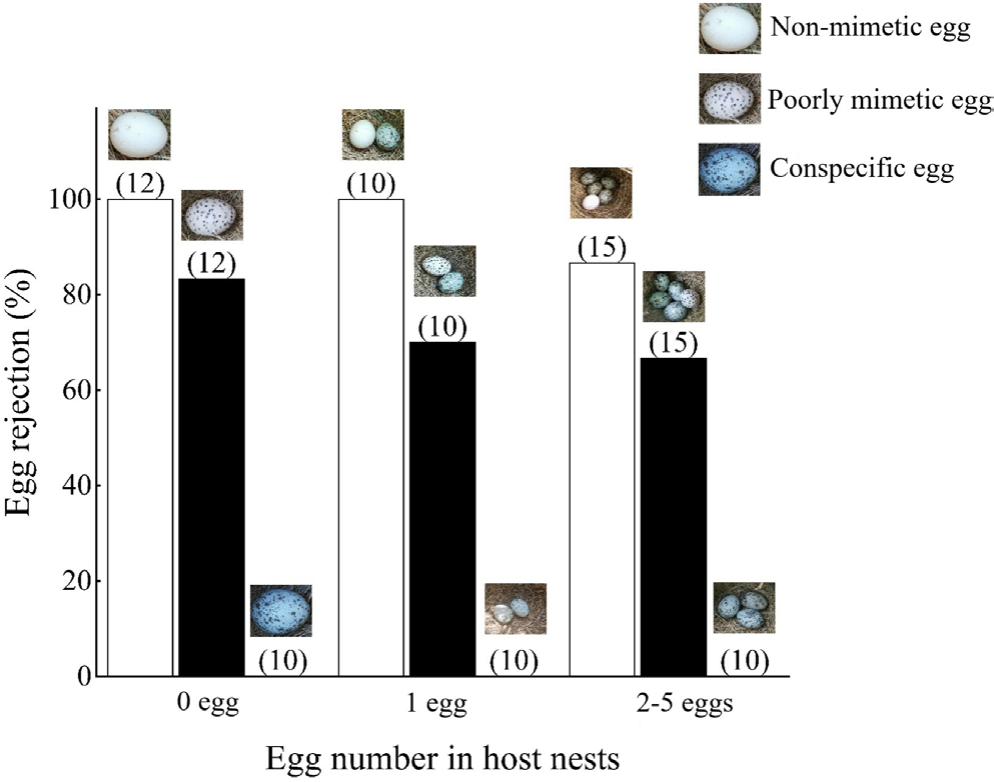
Relationship between rejection responses by the oriental reed warbler (including egg rejection and nest abandonment) to three different egg mimicry levels and the clutch size in the nest (number in parentheses refers to the sample size). 0 egg refers to the pre‐egg‐laying stage, 1 egg refers to the single‐host egg stage, 2–5 eggs refer to the multi‐host egg stage.

In the data (all raw data can be found in Table S1), nest desertion and egg ejections were summarized as egg rejection. Therefore, the rejection rate is defined as the number of nests exhibiting egg rejection behavior in each experimental group at each egg stage divided by the total number of nests in that experimental group (Table 1). In all the experiments (Table S2), the ORWs did not reject the highly mimetic eggs at all egg‐laying stages (Table 1, Figure 2). The rejection rates of poorly mimetic eggs across all egg‐laying stages were: (i) 83.3% (10/12, *n* = 12nests) during the pre‐egg‐laying stage; (ii) 70% (7/10, *n* = 10 nests) during the single‐host egg stage; and (iii) 66.7% (10/15, *n* = 15 nests) during the multiple‐host eggs stage. The nonmimetic eggs had 100% rejection rates during both the pre‐egg‐laying stage (12/12, *n* = 12 nests) and single‐host egg stage (10/10, *n* = 10 nests), decreasing to 86.7% at the multiple‐host egg stage (13/15, *n* = 15 nests) (Table 1).

**TABLE 1:** Classification of behavioral responses of the oriental reed warbler to treatment eggs (%). EE refers to egg ejection, EA refers to egg acceptance, and NA refers to nest abandonment.

Stage	Nonmimetic egg			Poorly mimetic egg			Highly mimetic egg		
	EE	EA	NA	EE	EA	NA	EE	EA	NA
Pre‐egg‐laying	75	0	25	75	16.7	8.3	0	100	0
Single‐host egg	80	0	20	70	30	0	0	100	0
Multiple‐host egg	86.7	13.3	0	66.7	33.3	0	0	100	0

We employed Bayesian‐GLMM analysis to examine the effects of experimental factors on egg rejection outcomes. The model included egg stages, experimental egg groups, the interaction between egg stages and experimental egg groups, and nest ID as a random intercept term to account for the relatedness of offspring within individuals (Table S2). Given the complete segregation of outcomes in the high‐simulation group (all acceptance), a strong prior was applied. Four Markov chains were run, each with 4000 iterations (including 2000 warm‐up), yielding a total of 8000 posterior samples. The Rhat statistic for all parameters was 1.00, with effective sample sizes exceeding 3700, indicating robust model convergence and reliable estimates. Statistical analyzes were performed using the brms package in R (v.4.3.0, 2023‐04‐21). Furthermore, all data visualization plots were drawn using the functional plotting software Origin 2022 (Origin Lab., Northampton, MA, USA).

## 3 Results

Bayesian analysis revealed that experimental treatments significantly influenced egg rejection outcomes (Table 2). Compared to the reference group (highly mimetic eggs), the odds ratio for poorly mimetic egg groups was 9.21 (95% CI [3.04, 28.79]), and for nonmimetic egg groups it was 24.53 (95% CI [7.55, 84.00]), indicating that the simulation level of experimental eggs significantly increased the probability of acceptance. In other words, host rejection rates for poorly mimetic eggs and nonmimetic eggs were significantly higher than those for highly mimetic eggs.

**TABLE 2:** Bayesian logistic regression model fixed effects parameter estimates.

	Parameter	Posterior mean	Std. error	95% CI	OR	OR 95% CI
Egg stages	Intercept	−1.33	0.5	[−2.32, −0.36]	0.26	[0.10, 0.70]
	Single‐host egg	−0.42	0.62	[−1.65, 0.79]	0.66	[0.19, 2.20]
	Multi‐host egg	−0.62	0.6	[−1.83, 0.54]	0.54	[0.16, 1.72]
Experimental groups	Poorly mimetic	2.22	0.58	[1.11, 3.36]	9.21	[3.04, 28.79]
	Non mimetic	3.2	0.62	[2.02, 4.43]	24.53	[7.55, 84.00]
Interactions	Single‐host egg × poorly mimetic	0.28	0.74	[−1.13, 1.72]	1.32	[0.32, 5.58]
	Multi‐host egg × poorly mimetic	0.35	0.7	[−1.00, 1.74]	1.42	[0.37, 5.70]
	Single‐host egg × non mimetic	0.99	0.83	[−0.60, 2.64]	2.69	[0.55, 14.02]
	Multi‐host egg × non mimetic	0.49	0.76	[−0.98, 1.96]	1.63	[0.38, 7.10]

*Note:* Reference groups are pre‐egg‐laying (Egg stages) and highly mimetic eggs (Experimental groups). Random intercept standard deviation = 0.31 (95% CI [0.01, 0.92]). Model classification accuracy = 88.5%.

However, the main effect of egg stages and its interaction with the experimental groups (egg simulation group) was both insignificant, with the 95% confidence intervals for all relevant parameters encompassing 1. This may indicate that the host's rejection rate of experimental eggs is unaffected by egg stage.

## 4 Discussion

Our results show that ORWs rejected most non‐ and poorly mimetic eggs even during the pre‐egg‐laying stage, while accepting all highly mimetic (conspecific) eggs. This pattern of significantly rejecting poorly mimetic and nonmimetic eggs while accepting highly mimetic (conspecific) eggs may be consistent with the memory‐based template hypothesis (Lotem et al. 1995; Hauber and Sherman 2001; Wang et al. 2015). However, the consistent acceptance of conspecific eggs across all breeding stages, including the pre‐laying stage when no own eggs are present for comparison, could also be explained by an uncertainty‐based acceptance mechanism (Davies 2000; Servedio and Hauber 2006; Ruiz‐Raya and Soler 2020). According to this view, hosts may accept eggs that closely resemble their own not because they match a memorized template, but because the cost of mistakenly rejecting their own egg outweighs the cost of accepting a parasitic one when uncertainty is high (Servedio and Hauber 2006; Ruiz‐Raya and Soler 2020).

In our study, the 100% acceptance rate of conspecific eggs—even in the absence of any reference egg—suggests that ORWs may employ a dual mechanism: an innate or experientially acquired template for initial discrimination, complemented by a context‐dependent acceptance threshold that reduces the risk of rejection errors (Hauber et al. 2006; Ruiz‐Raya and Soler 2020). Although no statistical difference analysis was conducted, it has changed that the decline we observed in rejection rates from the pre‐laying to multiple‐egg stages for poorly mimetic eggs gives credence to this hypothesis and may reflect a shift in the rejection threshold as the clutch develops—a phenomenon documented in other host species (Hauber et al. 2006; Molina‐Morales et al. 2014).

Therefore, while our data are consistent with the memory‐based template hypothesis, we cannot rule out the role of uncertainty‐driven acceptance, especially for highly mimetic eggs. The acceptance of conspecific eggs during the pre‐egg‐laying stage could reflect a strategy where hosts avoid the costly error of rejecting their own eggs (Davies and Brooke 1988; Ruiz‐Raya and Soler 2020). Future studies should aim to disentangle these mechanisms by experimentally manipulating perceived risk and uncertainty, for example, by varying the ratio of parasitic to host eggs or by testing responses to eggs along a continuous gradient of mimicry (Hanley et al. 2019; Ruiz‐Raya and Soler 2020). Furthermore, without comparing responses between naïve and experienced individuals—e.g., first‐year versus older breeders—we cannot definitively distinguish between innate and learned templates (Lotem et al. 1995; Moskát, Bán, and Hauber 2014). Future work should therefore incorporate age or experience‐based comparisons, or contrast populations with differing parasitism histories, to better elucidate the cognitive and experiential underpinnings of egg recognition in this system (Kuehn et al. 2016; Langmore et al. 2012).

If foreign eggs in the nests during the pre‐egg‐laying stage were simply disposed of through nest clearing behaviors, then this may be a nest sanitation and a form of pre‐adaptation to egg rejection (Rothstein 1975; Moskát et al. 2003; Yang et al. 2015; Wang et al. 2015). However, in our study, ORWs accept all highly mimetic (conspecific) eggs across the three egg stages (pre‐egg‐laying, single host egg, and multiple host eggs), suggesting that ORWs do not use the onset of laying (Davies 2000) for egg recognition, as they accept all highly mimetic eggs prior to the onset of their own laying. In addition, ORWs were also not simply disposing of eggs in their nests through nest sanitation (Moskát et al. 2003; Yang et al. 2015).

The consistent acceptance of highly mimetic conspecific eggs by Oriental reed warblers, even in the absence of their own eggs, raises the intriguing possibility that ORWs may not recognize their own eggs individually, but rather rely on a generalized phenotypic template of conspecific egg appearance. This contrasts with some other host species, such as the chaffinch ( *Fringilla coelebs* ), which can reject moderately mimetic conspecific eggs (Stokke et al. 2004), and the Australian reed warbler ( *Acrocephalus australis* ), which shows strong egg discrimination under certain parasitism contexts (Welbergen et al. 2001). The lack of rejection toward conspecific eggs in ORWs may reflect a perceptual or cognitive threshold beyond which eggs are accepted if they fall within the acceptable range of conspecific variation—a concept aligned with the optimal acceptance threshold hypothesis (Reeve 1989; Hanley et al. 2019). Recent studies using continuous gradients of egg traits (e.g., color, shape) have shown that hosts such as American robins ( *Turdus migratorius* ) exhibit graded rejection responses as model eggs deviate from natural appearance (Hauber et al. 2021; Fernandez‐Duque et al. 2025). We propose that future experiments using 3D‐printed egg models with systematically varied color, maculation, and shape could help identify the specific visual cues and threshold values that trigger egg rejection in ORWs. Such an approach would clarify whether ORWs use a single trait or a combination of traits for egg recognition, and whether their acceptance of conspecific eggs is due to a lack of discrimination ability or a high tolerance for phenotypic variation.

In conclusion, our study shows that this ORW population did not reject any conspecific eggs (highly mimetic) across all three egg stages, whereas at each egg stage (pre‐laying, single‐host egg, or multi‐host egg stage), they rejected the majority of nonmimetic and poorly mimetic eggs. ORWs may mainly use a memory‐based template as an egg discrimination mechanism against parasitism, meaning that they use innate or long‐term memory templates derived from previous reproduction attempts for egg recognition. The recognition template may be formed through observational learning or the gaining of experience. However, the consistent acceptance of eggs from the same species may also align with an uncertainty‐based acceptance strategy, which warrants further exploration in future research. Furthermore, rejection of most foreign eggs, but none of the conspecific eggs, at the pre‐egg‐laying stage without any references may be true egg recognition in response to parasitism, rather than as a behavioral response triggered by nest sanitation. Our study provides insights for a more comprehensive understanding of the recognition mechanism in ORWs as a defense against brood parasitism. Future studies should carry out additional research on the complex cognitive basis and multiple mechanisms for egg discrimination of ORWs in other geographical populations.

## Author Contributions

**Hanlin Yan:** data curation (equal), formal analysis (equal), investigation (equal), methodology (equal), visualization (equal), writing – original draft (equal). **Longwu Wang:** conceptualization (equal), funding acquisition (equal), resources (equal), supervision (equal), validation (equal), writing – review and editing (equal). **Wei Liang:** conceptualization (equal), funding acquisition (equal), supervision (equal), validation (equal), writing – review and editing (equal).

## Funding

This work was supported by the National Natural Science Foundation of China (nos. 32270526 and 32470513 to W.L., 32260253 to L.W.). H.Y. was supported by Guizhou Province Basic Research Program (Natural Sciences) Youth Guidance Project [Qiankehe Basic QN (2025) 259], and the High‐level Talent Research Start‐up Project of Liupanshui Normal University (LPSSYKYJJ202512).

## Ethics Statement

The study was conducted in compliance with the law of China. Experimental procedures in China were in accordance with the Animal Research Ethics Committee of Hainan Provincial Education Centre for Ecology and Environment, Hainan Normal University (no. HNECEE‐2012‐003) and Guizhou Normal University (No. GZNUECEE‐2021‐001).

## Conflicts of Interest

The authors declare no conflicts of interest.

## Supporting information


**Table S1:** All raw data used for this study with nest desertion and egg ejected being summarized as egg rejection.

**Table S2:** Data of egg experiments for the three breeding stages.

**Table S3:** Data of different egg mimicry for the egg experiments.

## Data Availability

Data used in this study are presented in Tables [Link], [Link], [Link] and can be found at https://figshare.com/s/5da6964e3f496a038092 (doi: 10.6084/m9.figshare.29552144).
